# Supplementary material for: MEILB2-BRME1 forms a V-shaped DNA clamp upon BRCA2-binding in meiotic recombination
Source: Nat Commun. 2024 Aug 2;15:6552. doi: 10.1038/s41467-024-50920-x (PMC11297322; doi:10.1038/s41467-024-50920-x)
Supplement: Supplementary file 1 — Supplementary Information [file 41467_2024_50920_MOESM1_ESM.pdf]

Supplementary Information

MEILB2-BRME1 forms a V-shaped DNA clamp upon BRCA2-binding in meiotic recombination

Manickam Gurusaran, Jingjing Zhang, Kexin Zhang, Hiroki Shibuya and Owen R. Davies

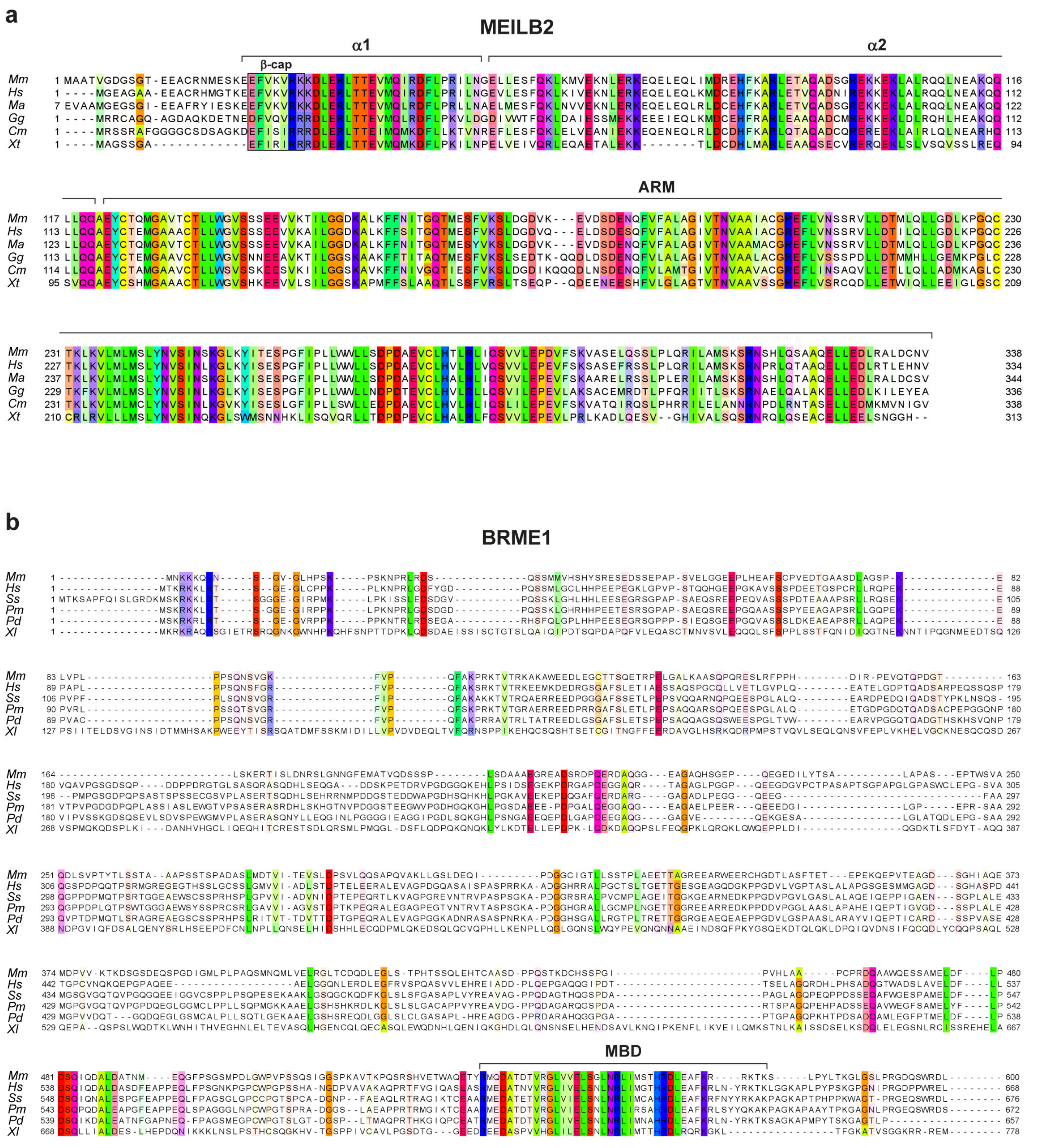

Supplementary Figure 1

Multiple sequence alignments of MEILB2 and BRME1.

(**a**) Multiple sequence alignment of MEILB2, highlighting the locations of the α1, α2 and ARM regions of the protein, in addition to the β-cap at the beginning of the α1 region. *Mus musculus* (*Mm*), *Homo sapiens* (*Hs*), *Mesocricetus auratus* (*Ma*), *Gallus gallus* (*Gg*), *Callorhinchus milii* (*Cm*), and *Xenopus tropicalis* (*Xt*). (**b**) Multiple sequence alignment of BRME1, highlighting the locations of the MEILB2-

binding domain (MBD). *Mus musculus* (*Mm*), *Homo sapiens* (*Hs*), *Sus scrofa* (*Ss*), *Physeter macrocephalus* (*Pm*), *Phyllostomus discolor* (*Pd*), and *Xenopus laevis* (*Xl*).

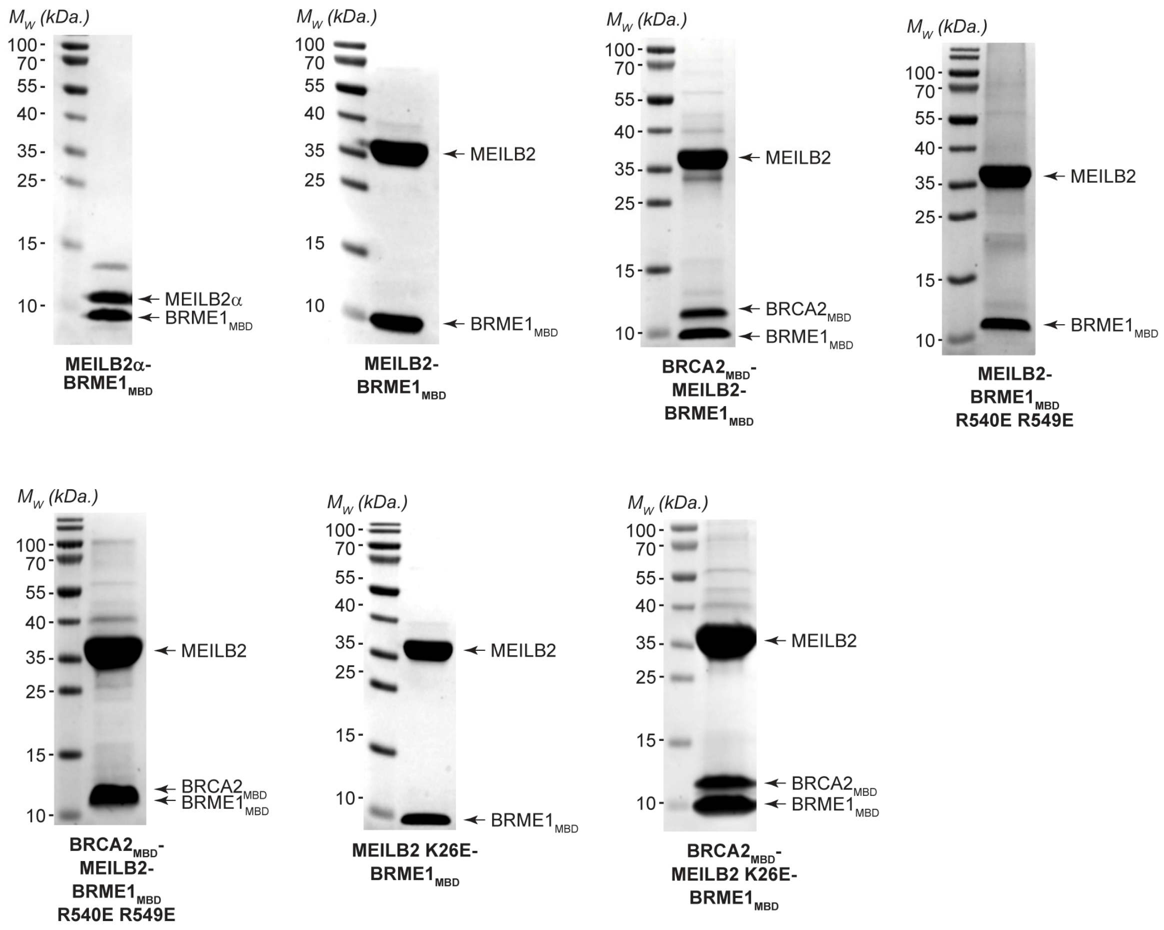

Supplementary Figure 2

SDS-PAGE of protein samples used in this study.

MEILB2α-BRME1$_{MBD}$ 2:2 core complex
2Fo-Fc map (1.5σ)

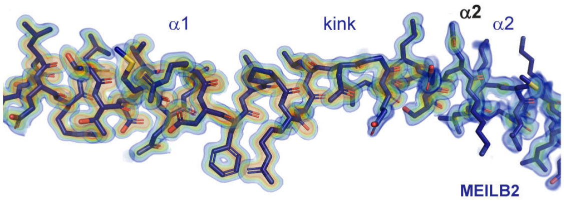

Supplementary Figure 3

Crystal structure of the MEILB2α-BRME1$_{MBD}$ 2:2 core complex.

2Fo-Fc electron density map of the MEILB2α-BRME1$_{MBD}$ 2:2 core structure (1.5σ), presented as a rainbow between 1.5σ (blue) and 3.5σ (red), superimposed on the refined crystallographic model.

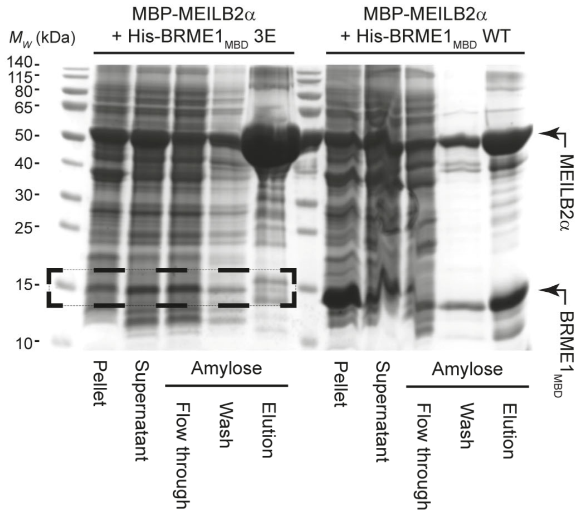

Supplementary Figure 4

Pull-down of the MEILB2α-BRME1$_{MBD}$ core complex.

Amylose pull-down of His-BRME1$_{MBD}$ wild-type (WT) and His-BRME1$_{MBD}$ V548E L555E I562E (3E) with MBP-MEILB2α following recombinant co-expression, corresponding to Figure 3b.

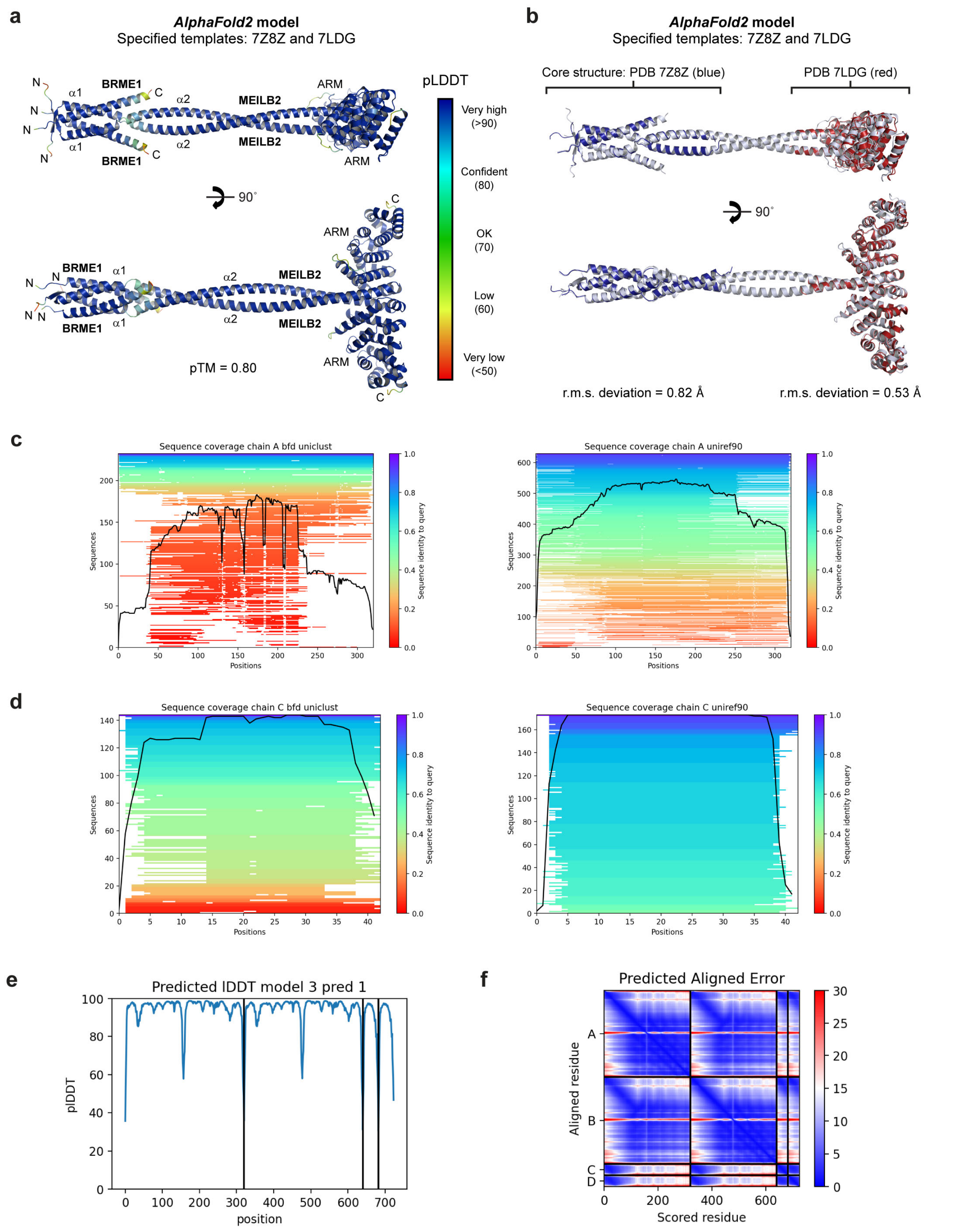

Supplementary Figure 5

***AlphaFold2* model of the full MEILB2-BRME1$_{MBD}$ 2:2 complex.**

(**a**,**b**) *AlphaFold2* model of the full MEILB2-BRME1$_{MBD}$ 2:2 complex, generated using the MEILB2α-BRME1$_{MBD}$ 2:2 structure reported herein (PDB accession 7Z8Z), and the MEILB2 ARM domain structure

(PDB accession 7LDG; Pendlebury et al., 2021), as the sole templates. Corresponds to Figure 4a. (**a**) MEILB2-BRME1$_{MBD}$ 2:2 model coloured according to predicted LDDT (pLDDT) scores, between blue (>90) and red (<50). (**b**) MEILB2-BRME1$_{MBD}$ 2:2 model (light blue), with the superimposed MEILB2α-BRME1$_{MBD}$ 2:2 crystal structure (dark blue; PDB accession 7Z8Z) and the MEILB2 ARM dimer crystal structure (red; PDB accession 7LDG) templates, showing r.m.s. deviations of 0.82 Å and 0.52 Å, respectively. (**c,d**) Representations of the multiple sequence alignments generated and used by *AlphaFold2*, showing the number of sequences and sequence identity against the position along the (**c**) MEILB2 and (**d**) BRME1 query sequences. (**e**) Predicted LDDT (pLDDT) scores shown for each amino-acid of the two MEILB2 and two BRME1 chains. (**f**) Predicted aligned error scores between each amino-acid of the two MEILB2 and two BRME1 chains, between blue (low error) and red (high error).

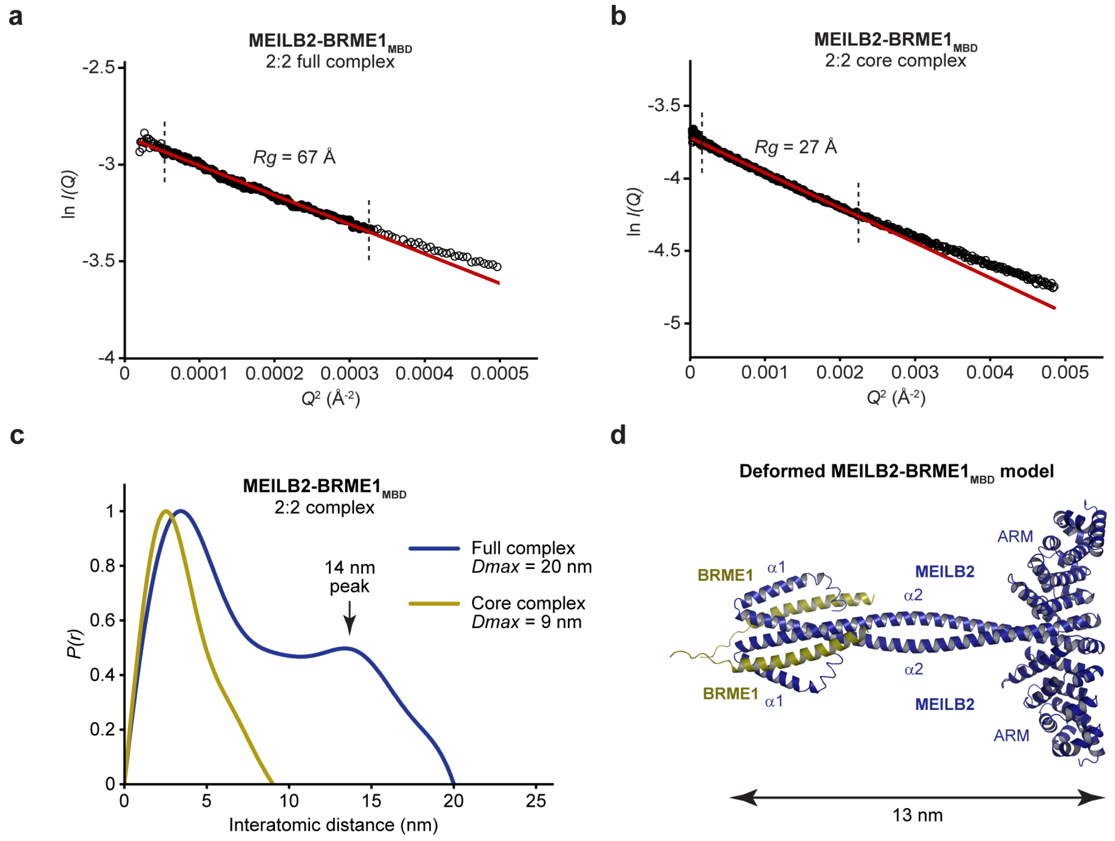

Supplementary Figure 6

SEC-SAXS analysis of the MEILB2-BRME1$_{MBD}$ 2:2 complex.

(**a**,**b**) SAXS Guinier analysis of the (**a**) MEILB2-BRME1$_{MBD}$ 2:2 full complex and (**b**) MEILB2α-BRME1$_{MBD}$ 2:2 core complex to determine the radius of gyration (*Rg*); linear fits are shown in red, with the fitted data range highlighted in black and demarcated by dashed lines. The *Q.Rg* values were < 1.3 and *Rg* values were calculated as (**a**) 67 Å and (**b**) 27 Å. (**c**) SAXS *P(r)* interatomic distance distributions of the MEILB2-BRME1$_{MBD}$ 2:2 full (blue) and MEILB2α-BRME1$_{MBD}$ 2:2 core (yellow) complexes, showing maximum dimensions (*Dmax*) of 20 nm and 9 nm, respectively. The *P(r)* distribution for the full complex includes a peak at 14 nm, indicating that domains within the structure are separated by this distance. (**d**) Deformed version of the MEILB2-BRME1$_{MBD}$ *AlphaFold2* model, used as a control for fitting to SAXS scattering data. This was created by introducing a turn at the kink between MEILB2's α1 and α2 helices, such that α1 helices and BRME1 chains pack against α2 helices. The deformed model has a similar structure but is shorter than the original model (13 nm rather than 18 nm).

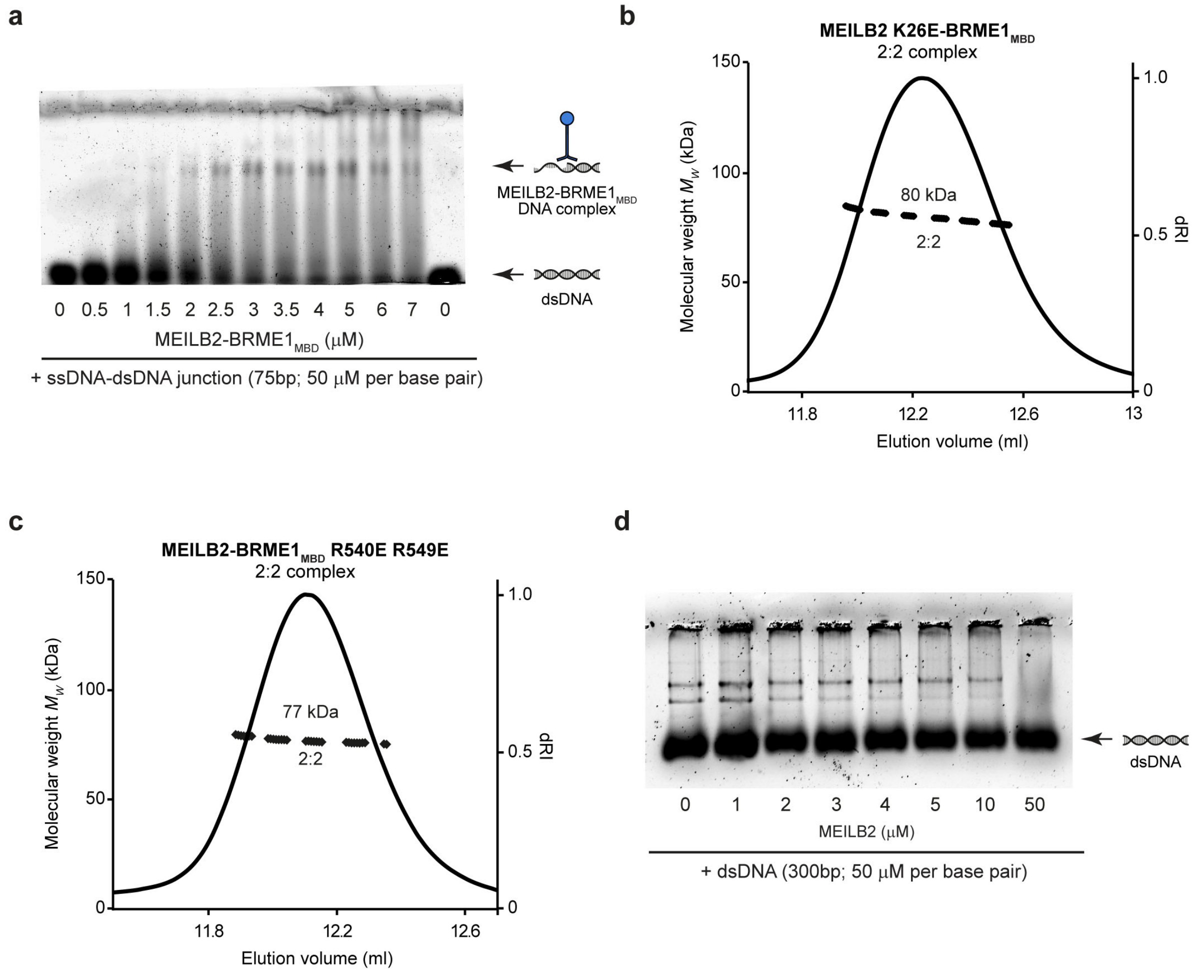

Supplementary Figure 7

DNA-binding of the MEILB2-BRME1$_{MBD}$ 2:2 complex.

(**a**) EMSA analysing the ability of MEILB2-BRME1$_{MBD}$ (at molecular concentrations indicated) to interact with an ssDNA/dsDNA junctional substrate (75 base pairs), at a concentration of 50 μM per base pair. (**b,c**) SEC-MALS analysis of MEILB2-BRME1$_{MBD}$ with (**b**) MEILB2 K26E and (**c**) BRME1 R540E R549E mutations, demonstrating the formation of 2:2 species of 80 kDa and 77 kDa, respectively (theoretical *Mw* – 82 kDa). (**d**) EMSA analysing the ability of MEILB2 (at molecular concentrations indicated) to interact with dsDNA (300 base pairs), at a concentration of 50 μM per base pair.

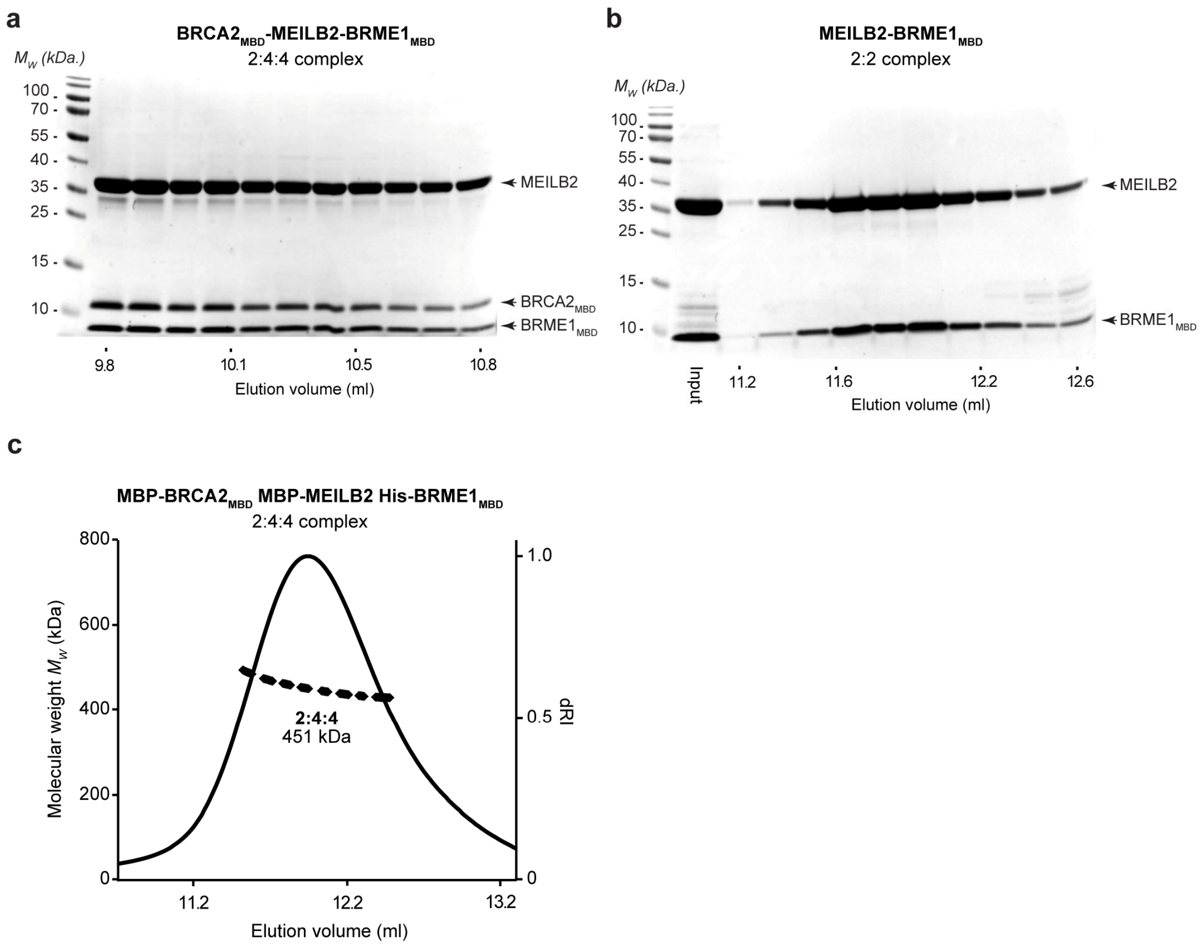

Supplementary Figure 8

BRCA2$_{MBD}$-MEILB2-BRME1$_{MBD}$ forms a 2:4:4 ternary complex.

(**a,b**) SDS-PAGE of elution fractions of the (**a**) BRCA2$_{MBD}$-MEILB2-BRME1$_{MBD}$ 2:4:4 complex and the (**b**) MEILB2-BRME1$_{MBD}$ 2:2 complex, corresponding to the SEC-MALS data shown in Figure 6a. (**c**) SEC-MALS analysis of MBP-BRCA2$_{MBD}$ MBP-MEILB2 His-BRME1$_{MBD}$, demonstrating the formation of a 2:4:4 species of 451 kDa (theoretical *Mw* – 454 kDa).

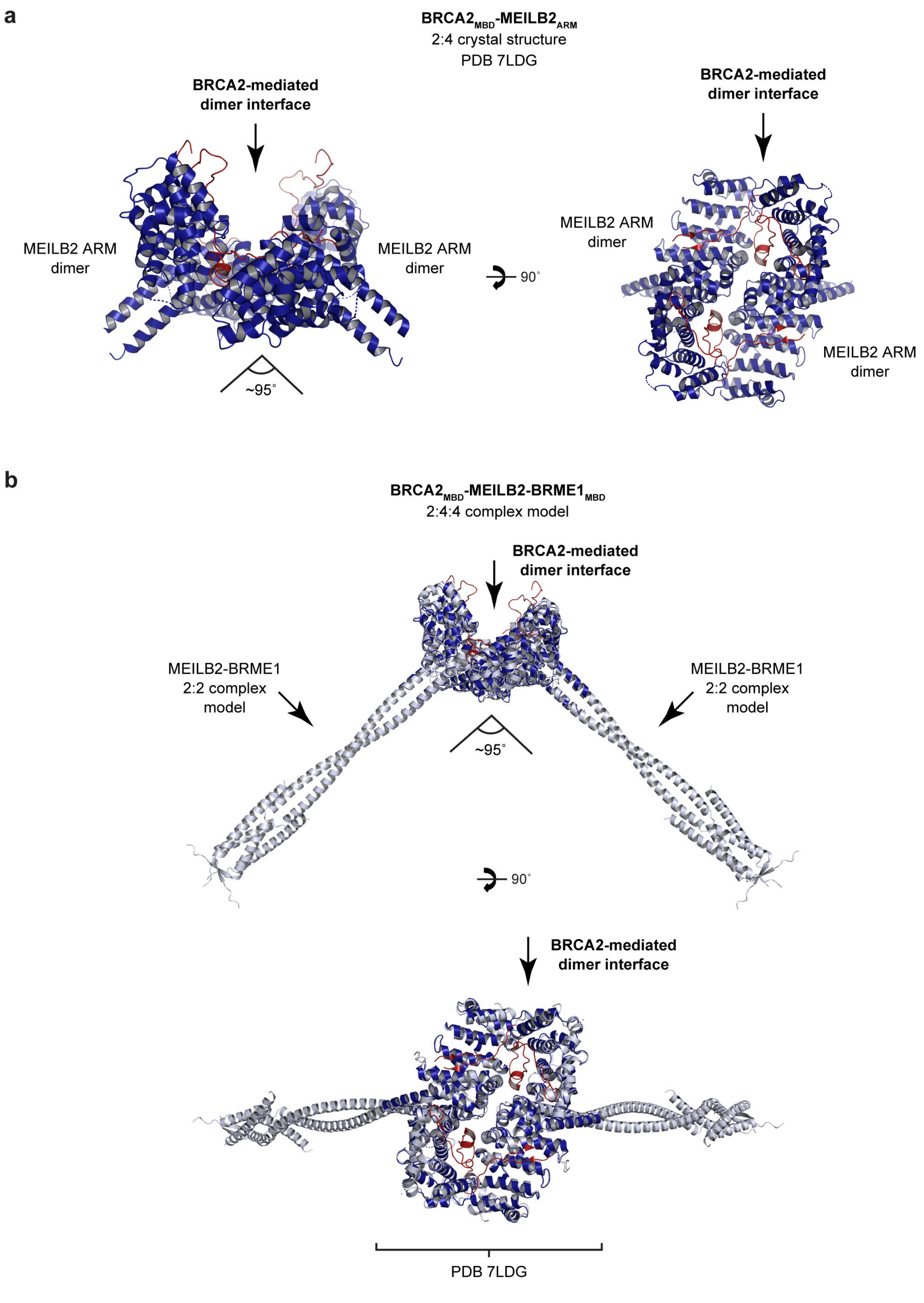

Supplementary Figure 9

Model of the BRCA2$_{MBD}$-MEILB2-BRME1$_{MBD}$ 2:4:4 ternary complex.

(**a**) Crystal structure of the BRCA2$_{MBD}$-MEILB2 ARM 2:4 complex, showing how BRCA2$_{MBD}$ mediates the dimerization of two opposing MEILB2 ARM dimers, which held at approximately 95° to one another

(PDB accession 7LDG; Pendlebury et al., 2021). (**b**) Model of the $BRCA2_{MBD}$-MEILB2-$BRME1_{MBD}$ 2:4:4 ternary complex (corresponding to Figure 6b), generated by docking two models of the full MEILB2-$BRME1_{MBD}$ 2:2 complex onto the $BRCA2_{MBD}$-MEILB2 ARM 2:4 crystal structure. In the resultant assembly, $BRCA2_{MBD}$ mediates dimerization of the two constituent MEILB2-$BRME1_{MBD}$ 2:2 complex, in which their limbs are held at approximately 95° to one another.

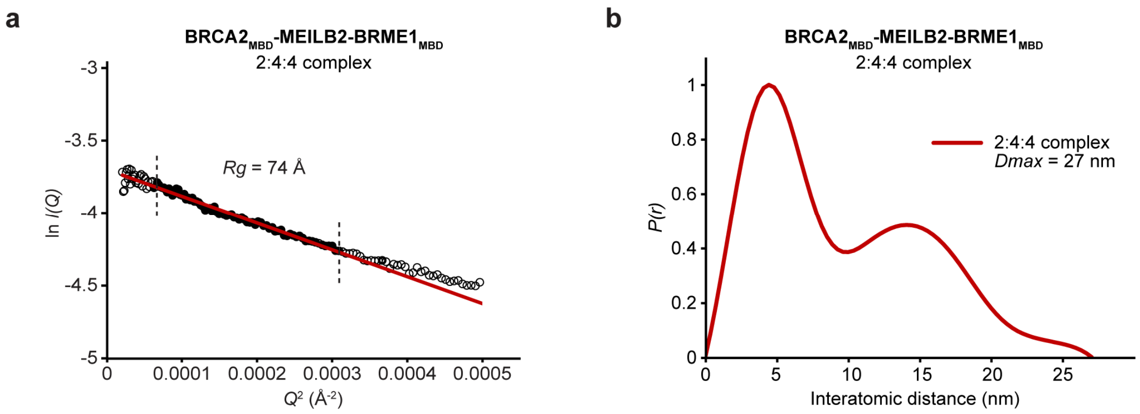

Supplementary Figure 10

SEC-SAXS analysis of the BRCA2$_{MBD}$-MEILB2-BRME1$_{MBD}$ 2:4:4 ternary complex.

(**a**) SAXS Guinier analysis of the BRCA2$_{MBD}$-MEILB2-BRME1$_{MBD}$ 2:4:4 ternary complex to determine the radius of gyration (*Rg*); the linear fit is shown in red, with the fitted data range highlighted in black and demarcated by dashed lines. The *Q.Rg* values were < 1.3 and *Rg* was calculated as 74 Å. (**b**) SAXS *P(r)* interatomic distance distribution of the BRCA2$_{MBD}$-MEILB2-BRME1$_{MBD}$ 2:4:4 ternary complex, showing a maximum dimension (*Dmax*) of 27 nm.

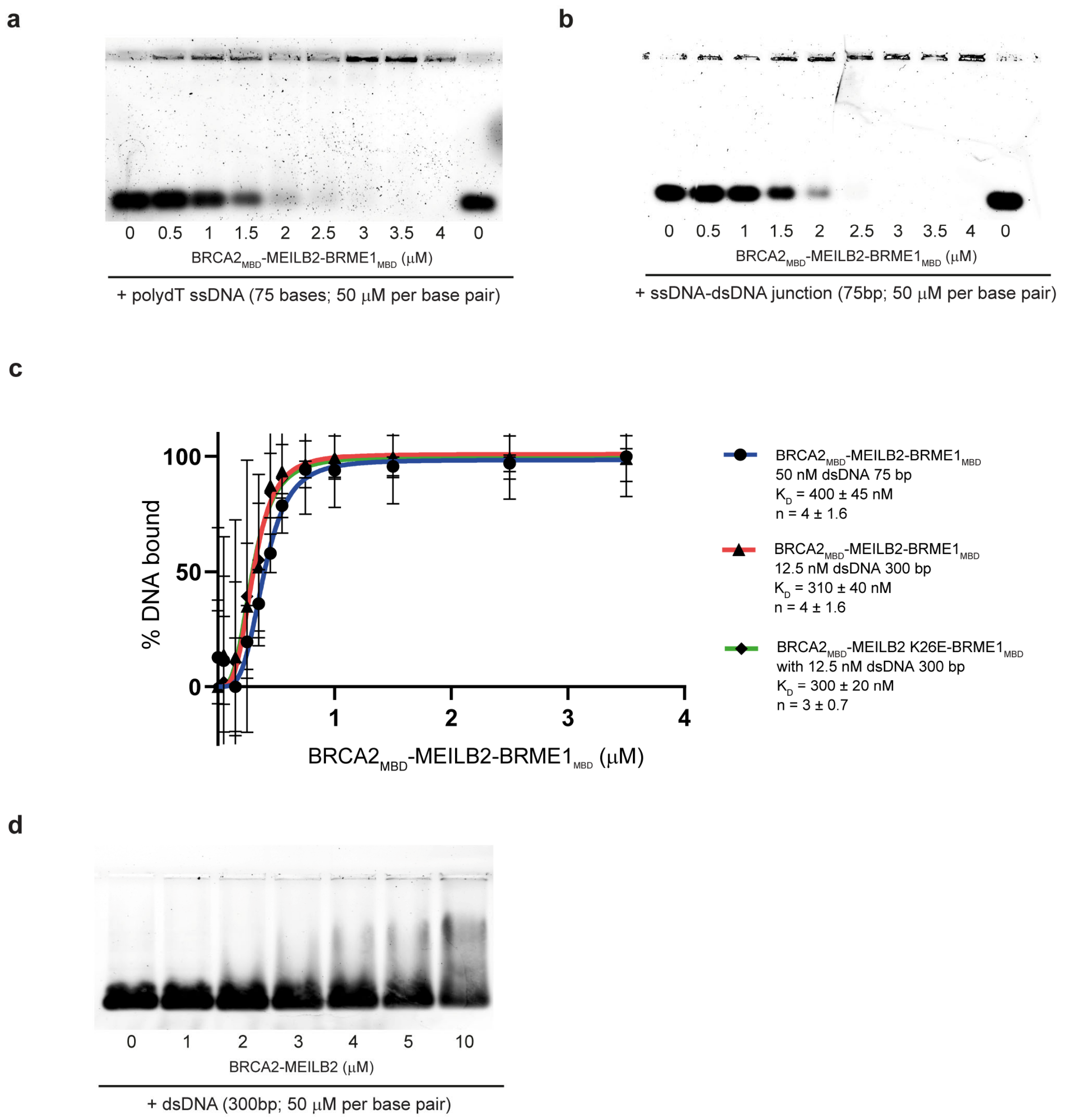

Supplementary Figure 11

DNA-binding of the BRCA2$_{MBD}$-MEILB2-BRME1$_{MBD}$ 2:4:4 ternary complex.

(**a,b**) EMSAs analysing the ability of BRCA2$_{MBD}$-MEILB2-BRME1$_{MBD}$ (at molecular concentrations indicated) to interact with (**a**) polydT ssDNA and (**b**) an ssDNA/dsDNA junctional substrate (75 base pairs), at a concentration of 50 µM per base pair. (**c**) Quantification of DNA-binding by BRCA2$_{MBD}$-MEILB2-BRME1$_{MBD}$ and BRCA2$_{MBD}$-MEILB2 K26E-BRME1$_{MBD}$ through densitometry of EMSAs performed using 50 nM (per molecule) FAM-dsDNA (75 base pairs; blue and green) and 12.5 nM (per molecule) FAM-dsDNA (300 base pairs; red). Plots, K_D and Hill coefficient (n) values were determined

by fitting data to the Hill equation and are quoted within a 95% confidence interval; data are presented as mean values, with error bars indicating standard error, *n* = 3 EMSAs. (**d**) EMSA analysing the ability of $BRCA2_{MBD}$-MEILB2 (at molecular concentrations indicated) to interact with dsDNA (300 base pairs), at a concentration of 50 μM per base pair.

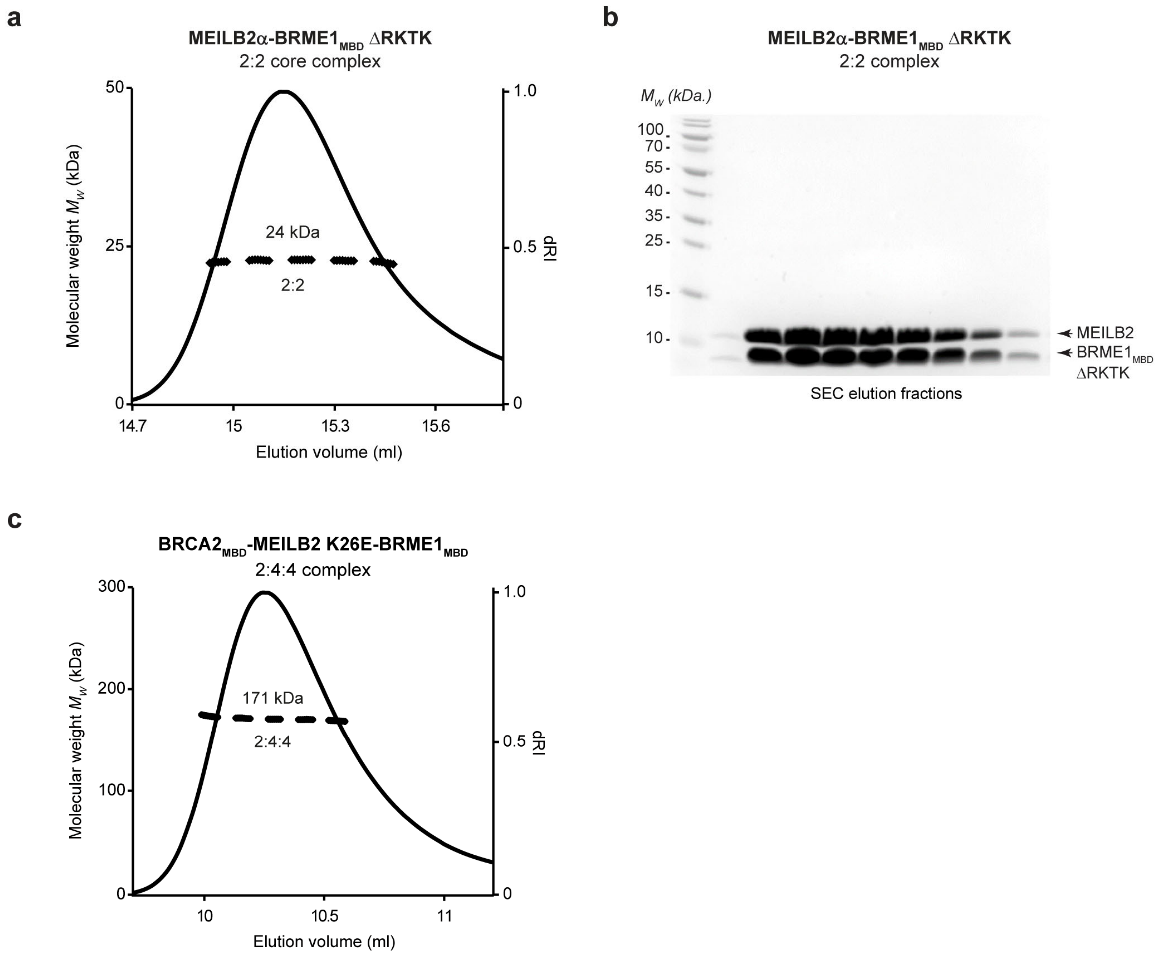

Supplementary Figure 12

BRCA2$_{MBD}$-MEILB2-BRME1$_{MBD}$ complex formation by mutants and truncations.

(**a**) SEC-MALS analysis of MEILB2α-BRCA2$_{MBD}$ ΔRKTK, demonstrating the formation of a 2:2 species of 24 kDa (theoretical *Mw* – 24 kDa). (**b**) SDS-PAGE of elution fractions of MEILB2α-BRCA2$_{MBD}$ ΔRKTK corresponding to the SEC-MALS analysis shown in panel a. (**c**) SEC-MALS analysis of BRCA2$_{MBD}$-MEILB2 K26E-BRME1$_{MBD}$, demonstrating the formation of a 2:4:4 species of 171 kDa (theoretical *Mw* – 176 kDa).

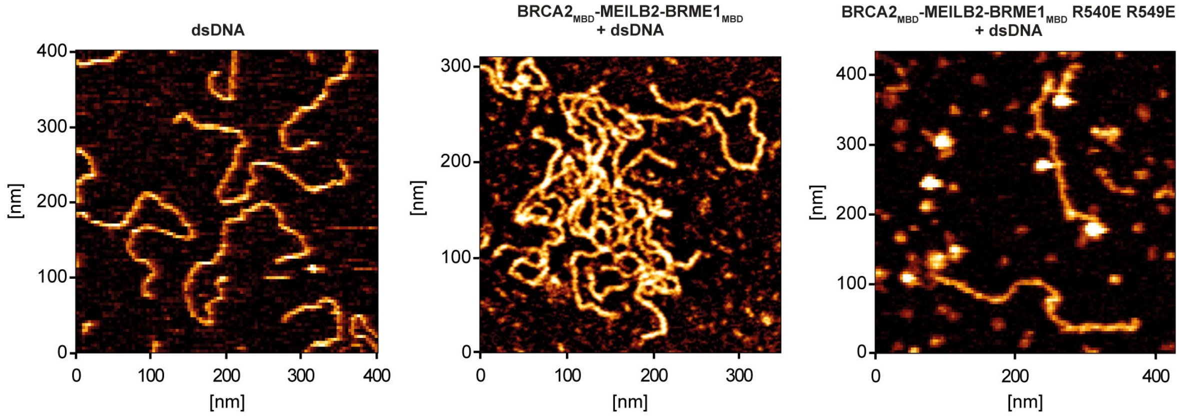

Supplementary Figure 13

Atomic force microscopy of BRCA2$_{MBD}$-MEILB2-BRME1$_{MBD}$ complexes with dsDNA.

Atomic force microscopy images of linear dsDNA (1.3 kbp) in isolation (left), upon binding by BRCA2$_{MBD}$-MEILB2-BRME1$_{MBD}$ complex (middle), and in the presence of the BRCA2$_{MBD}$-MEILB2-BRME1$_{MBD}$ harbouring the R540E R549E mutation (right).

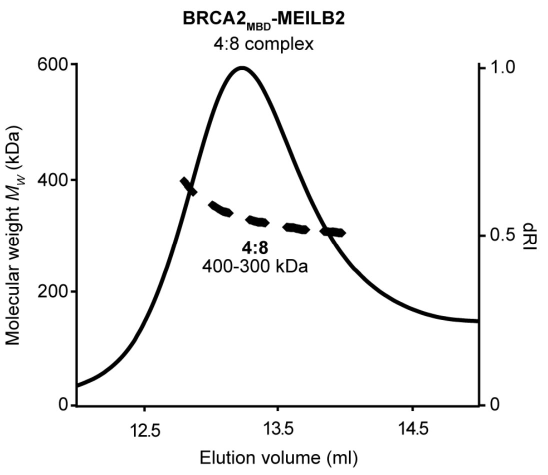

Supplementary Figure 14

SEC-MALS analysis of BRCA2$_{MBD}$-MEILB2.

SEC-MALS analysis of BRCA2$_{MBD}$-MEILB2, demonstrating 300-400 kDa species that is consistent with formation of a 4:8 complex (theoretical *Mw* – 313 kDa).

Supplementary Table 1

SEC-SAXS data table.

(*a*) Sample details			
	MEILB2-BRME1$_{MBD}$ 2:2 full complex	MEILB2-BRME1$_{MBD}$ 2:2 core complex	BRCA2$_{MBD}$-MEILB2-BRME1$_{MBD}$ 2:4:4 complex
Organism	*M. Musculus*	*M. Musculus*	*M. Musculus*
Description: sequence (including Uniprot ID + uncleaved tags), bound ligands/modifications, *etc.*	>BRME1; Q6DIA7<br>GSMRMQDAT<br>DTVRGLV<br>VELSGLNR<br>LIMSTHRD<br>LEAFKRRK<br>TK<br>>MEILB2; Q9D4G2<br>GSMEEFVK<br>VRKKDLER<br>LTTEVMQI<br>RDFLPRIL<br>NGELLESF<br>QKLKMVEK<br>NLERKEQE<br>LEQLIMDR<br>EHFKARLE<br>TAQADSGR<br>EKKEKLAL<br>RQQLNEAK<br>QQLLQQAE	>BRME1; Q6DIA7<br>GSMRMQDA<br>TDTVRGLV<br>VELSGLNR<br>LIMSTHRD<br>LEAFKRRK<br>TK<br>>MEILB2; Q9D4G2<br>GSMEEFVK<br>VRKKDLER<br>LTTEVMQI<br>RDFLPRIL<br>NGELLESF<br>QKLKMVEK<br>NLERKEQE<br>LEQLIMD	>BRCA2; P97929<br>GSMPIKRSL<br>LNEFDRIIE<br>SKGKSLTP<br>SKSTPDGT<br>VKDRSLFT<br>HHMSLEPV<br>TCGPF<br>>BRME1; Q6DIA7<br>GSMRMQDA<br>TDTVRGLV<br>VELSGLNR<br>LIMSTHRD<br>LEAFKRRK<br>TK<br>>MEILB2; Q9D4G2<br>GSMEEFVK<br>VRKKDLER<br>LTTEVMQI<br>RDFLPRIL<br>NGELLESF<br>QKLKMVEK

YCTQMGAV

TCTLLWGV

SSSEEVVK
TILGGDKA

LKFFNITG

QTMESFVK

SLDGDVKE

VDSDENQF

VFALAGIV

TNVAAIAC

GREFLVNS

SRVLLDTM

LQLLGDLK

PGQCTKLK

VLMLMSLY

NVSINSKG

LKYITESP

GFIPLLWW

LLSDPDAE

VCLHTLRL

IQSVVLEP

DVFSKVAS

ELQSSLPL

QRILAMSK

SRNSHLQS

AAQELLED

LRALDCNV

NLERKEQE LEQLIMDR

EHFKARLE

TAQADSGR

EKKEKLAL

RQQLNEAK

QQLLQQAE

YCTQMGAV

TCTLLWGV

SSSEEVVK

TILGGDKALKFFNITG

QTMESFVK

SLDGDVKE

VDSDENQF

VFALAGIV

TNVAAIAC

GREFLVNS

SRVLLDTM

LQLLGDLK

PGQCTKLK

VLMLMSLY

NVSINSKG

LKYITESP

GFIPLLWW

LLSDPDAE

VCLHTLRL

IQSVVLEP

DVFSKVAS

ELQSSLPL

QRILAMSK

			SRNSHLQS AAQELLED LRALDCNV
Extinction coefficient ε (280 nm; M^{-1} cm^{-1})	41940	0	83880
Molecular mass *M* from chemical composition (Da)	81861.0	24885.08	175871.9
For SEC-SAS, loading volume/concentration, (mg ml^{-1})<br>injection volume (μl), flow rate (ml min^{-1})	11 mg ml^{-1}<br>100 μl<br>0.5 ml min^{-1}	10 mg ml^{-1}<br>100 μl<br>0.5 ml min^{-1}	8 mg ml^{-1}<br>100 μl<br>0.5 ml min^{-1}
Solvent composition and source	20 mM HEPES pH 7.5, 150 mM KCl, 2 mM DTT	20 mM HEPES pH 7.5, 150 mM KCl, 2 mM DTT	20 mM HEPES pH 7.5, 500 mM KCl, 2 mM DTT

(*b*) SAS data collection parameters

Source, instrument and description or reference: Beamline B21 of Diamond Light Source

Wavelength (Å): 0.99987 Å

Beam geometry (size, sample-to-detector distance): 1.1 x 0.2 mm cross-section, detector distance 4.014 m

q-measurement range ($Å^{-1}$ or nm^{-1}): $Å^{-1}$

Exposure time, number of exposures: 3.0 s exposure time; 1200 exposures

Sample temperature (ºC): 22ºC

(*c*) Software employed for SAS data reduction, analysis and interpretation

SAS data reduction to sample–solvent scattering: *ScÅtter* 3.0

Basic analyses: Guinier, *P*(*r*), scattering particle volume (*e.g.* Porod volume V_P or volume of correlation V_c): *PRIMUS*

Atomic structure modelling (homology, rigid body, ensemble) *CRYSOL*

Molecular graphics: *PyMOL*

(*d*) Structural parameters

Guinier Analysis	MEILB2-BRME1$_{MBD}$ 2:2 full complex	MEILB2-BRME1$_{MBD}$ 2:2 core complex	BRCA2$_{MBD}$-MEILB2-BRME1$_{MBD}$ 2:4:4 complex
I(0) (cm^{-1})	0.057	0.024	0.012

R_g (Å)	67	27	74
q-range ($Å^{-1}$)	0.005 – 0.3	0.005 – 0.3	0.005 – 0.2
M from *I*(0) (ratio to expected value)	1.33	1.15	1.03
P(*r*) analysis	MEILB2-BRME1$_{MBD}$ 2:2 full complex	MEILB2-BRME1$_{MBD}$ 2:2 core complex	BRCA2$_{MBD}$-MEILB2-BRME1$_{MBD}$ 2:4:4 complex
I(0) (cm^{-1})	0.057	0.024	0.012
R_g (Å)	68	28	75
d_{max} (Å)	200	90	270
q-range ($Å^{-1}$)	0.005 – 0.3	0.005 – 0.3	0.005 – 0.2
Quality-of-fit parameter	0.6840	0.6760	0.5974
Volume (V_P)	454218	36165	426731

(e) Atomistic modelling

	MEILB2-BRME1$_{MBD}$ 2:2 full complex	MEILB2-BRME1$_{MBD}$ 2:2 core complex	BRCA2$_{MBD}$-MEILB2-BRME1$_{MBD}$ 2:4:4 complex
Method	*CRYSOL* – fit to the 2:2 complex model	*CRYSOL* – fit to the 2:2 crystal structure	*CRYSOL* – fit to the 2:4:4 complex model
q-range for fitting	0.005 – 0.3	0.005 – 0.3	0.005 – 0.2
χ^2 value/range	2.96	2.50	1.36
